# Knowledge and Response to the COVID-19 Pandemic in People With Severe Mental Illness in Bangladesh and Pakistan: A Cross-Sectional Survey

**DOI:** 10.3389/fpsyt.2022.785059

**Published:** 2022-02-14

**Authors:** Sukanya Rajan, Lewis W. Paton, Asiful Haidar Chowdhury, Gerardo A. Zavala, Faiza Aslam, Rumana Huque, Humaira Khalid, Pratima Murthy, Asad T. Nizami, Krishna Prasad Muliyala, David Shiers, Najma Siddiqi, Jan R. Boehnke

**Affiliations:** ^1^Department of Psychiatry, National Institute of Mental Health and Neurosciences (NIMHANS), Bangalore, India; ^2^Department of Health Sciences, University of York, York, United Kingdom; ^3^ARK Foundation, Dhaka, Bangladesh; ^4^Institute of Psychiatry, Benazir Bhutto Hospital, Rawalpindi, Pakistan; ^5^Greater Manchester Mental Health National Health Service Trust, Trust Headquarters, Manchester, United Kingdom; ^6^Division of Psychology and Mental Health, The University of Manchester, Manchester, United Kingdom; ^7^Primary Care and Health Sciences, Keele University, Newcastle, United Kingdom; ^8^Hull York Medical School, York, United Kingdom; ^9^Bradford District Care National Health Service Foundation Trust, Shipley, United Kingdom; ^10^School of Health Sciences, University of Dundee, Dundee, United Kingdom

**Keywords:** COVID-19, severe mental illness (SMI), epidemiology, knowledge, practices

## Abstract

**Background:**

People with severe mental illnesses (SMIs) are likely to face disproportionate challenges during a pandemic. They may not receive or be able to respond to public health messages to prevent infection or to limit its spread. Additionally, they may be more severely affected, particularly in low- and middle-income countries.

**Methods:**

We conducted a telephone survey (May–June 2020) in a sample of 1,299 people with SMI who had attended national mental health institutes in Bangladesh and Pakistan before the pandemic. We collected information on top worries, socioeconomic impact of the pandemic, knowledge of COVID-19 (symptoms, prevention), and prevention-related practices (social distancing, hygiene). We explored the predictive value of socio-demographic and health-related variables for relative levels of COVID-19 knowledge and practice using regularized logistic regression models.

**Findings:**

Mass media were the major source of information about COVID-19. Finances, employment, and physical health were the most frequently mentioned concerns. Overall, participants reported good knowledge and following advice. In Bangladesh, being female and higher levels of health-related quality of life (HRQoL) predicted poor and better knowledge, respectively, while in Pakistan being female predicted better knowledge. Receiving information from television predicted better knowledge in both countries. In Bangladesh, being female, accessing information from multiple media sources, and better HRQoL predicted better practice. In Pakistan, poorer knowledge of COVID-19 prevention measures predicted poorer practice.

**Conclusion:**

Our paper adds to the literature on people living with SMIs and their knowledge and practices relevant to COVID-19 prevention. Our results emphasize the importance of access to mass and social media for the dissemination of advice and that the likely gendered uptake of both knowledge and practice requires further attention.

## Introduction

The global Coronavirus (COVID-19) pandemic has affected almost all aspects of life for most of the world's population (1) including low- and middle-income countries (LMICs) in South Asia (2). Since March 2020, some form of lockdown and social distancing measures have been mandated by most countries in the region (3) in attempts to limit the spread of the disease with potentially serious consequences for people's livelihoods and food security (1), compounding the fears, anxiety, and stress caused by the disease itself (2).

There is emerging evidence that people with severe mental illness (SMI) may be disproportionately affected by both COVID-19 infection and the measures to limit the outbreak (4–6). For example, in a secondary analysis of electronic health records from the United States, people with recently diagnosed schizophrenia showed an elevated risk of COVID-19 infection (7). The higher prevalence of comorbid chronic physical health conditions and health risk behaviors [e.g., diabetes and smoking (8, 9)] in this population increases vulnerability to contracting the infection and to more severe adverse outcomes of COVID-19, including increased mortality (9, 10). Socio-economic disparities and poorer access to healthcare for people with SMI may further contribute to these increased risks (11); and people with SMI are likely to be especially vulnerable to economic hardships and social isolation associated with lockdown measures because of pre-pandemic poverty and limited social networks (12). Challenges to maintaining quality care for patients living with SMIs including how to implement preventive measures and outbreaks of COVID-19 on psychiatric wards have been widely documented (5, 13–15). Additionally, past research on emotional and coping responses in disaster and epidemic contexts suggests an increased risk for a heightened stress response in this population with a range of potentially negative sequelae (16). Finally, cognitive impairment associated with some SMIs and poor risk awareness are recognized factors that may prevent people with SMI from following preventative measures and further increase their risk of infection (17, 18).

Studies investigating knowledge and practices supporting the prevention of infection and spread report mixed results. For example, in Pakistan among the general population both inadequate knowledge and suboptimal practices (19) as well as adequate knowledge and positive attitude (20) have been found. Respondents in Bangladesh had a high level of knowledge and positive attitudes toward the COVID-19 guidelines (21). Findings from a review on knowledge, attitudes, and practice during the pandemic indicates good knowledge, optimistic attitudes, and good practice among the respondents from China, Italy, Iran, Jordan, the US, and the UK (22). General population studies of the impact of COVID-19 typically fail to reflect the perspectives of people with SMI either by explicitly excluding them in eligibility criteria, or by virtue of methods such as online surveys which disadvantage this population (23, 24). It is therefore important to understand the impact of the pandemic on this population and whether people with SMI receive, understand, and follow public health advice aiming to curb the spread of the infection and to keep individuals safe. Therefore, we sought to describe the pandemic-related experiences of people with SMI, including top concerns, impact on food, financial security and health and knowledge and behaviors relevant to COVID-19, and their associations with demographic and socioeconomic variables and mental health. The main question for the present study is whether knowledge about and practices relevant to COVID-19 are associated with socioeconomic variables and mental health.

## Materials and Methods

### Design and Population

We conducted a cross-sectional survey of health, health risk behaviors, and healthcare use in people with SMI in three South Asian countries before the pandemic started: the IMPACT study [ISRCTN registry: 88485933; (8, 25)].

Adults aged 18 years and above with SMI [schizophrenia, bipolar disorder, schizoaffective disorder, and depression with psychotic symptoms; clinician-diagnosed and confirmed using the international neuropsychiatric interview; MINI version 6.0 (26)], attending three national institutes of mental health (in Bangladesh, India, and Pakistan) between July 2019 and March 2020 were recruited to the IMPACT survey (25). Stratified sampling was used to recruit a sample comprising 80% outpatients and 20% inpatients, reflecting the service usual case mix proportion of inpatients and outpatients (25). The protocol provides further details including the justification of the sample size (25).

Here we are reporting results from a follow-up data collection after start of the pandemic (ISRCTN registry: 15571919). We conducted a cross-sectional telephone survey among the original participants who had provided consent to be contacted for future research.

### Recruitment, Consent, and Data Collection

Trained researchers contacted potential participants by telephone during May 6th and June 17th, 2020, to explain the study purpose and procedures assess capacity to consent and seek verbal consent. Information about the study was also made available online. Participants were informed of their right to withdraw at any time without giving a reason. In case of non-response, three further attempts to contact were made, after which the participant was deemed not contactable.

Data were collected via a telephone interview and recorded in a tablet device using Qualtrics (25). Each participant took around 40–60 min to answer the questions via phone.

### Ethics

The study received ethics approval from the National Center for Injury Prevention and Research, Bangladesh (CIPRB/ERC2018/003), National Bioethics Committee Pakistan (NBC-413/19/262) and the Research Governance Committee, Department of Health Sciences, University of York (RGC13-06-19). Due to a delay in ethics and regulatory approval, no data were collected at the Indian partner site at this time.

### Measures

The questions covering Knowledge, Behaviors, Information sources, and Experiences were developed by the team, drawing on previous work on the Middle East Respiratory Syndrome Coronavirus (27) and emerging best-practice/governmental guidelines in March/April 2020 to develop these questions (28, 29). The full surveys, including the newly developed questions, were trialed with nine patients in Bangladesh and five patients in Pakistan.

#### Knowledge of COVID-19 Symptoms and Its Prevention

We presented respondents with thirteen correct and incorrect statements about symptoms (seven) and control measures (six statements) and asked participants if they agreed or disagreed with them.

#### Behaviors to Limit Spread of COVID-19

Participants were asked if they practiced nine pandemic control measures, such as social distancing, wearing face masks, and increased hygiene measures.

#### Information Sources

We asked about the sources of information the participants were using to receive relevant information about the pandemic.

#### Experience of COVID-19

This was explored with questions about participants' own and their household's experiences of COVID-19 symptoms, testing and diagnosis, and where relevant, receipt of treatments for COVID-19.

#### Mental Health

The Patient Health Questionnaire [PHQ-9 (30); in Bangladesh Cronbach-α = 0.83 with a 95% confidence interval (95%-CI, 500 bootstrap samples) from 0.81 to 0.85 as an estimate of the score's reliability, *N* = 845; and Cronbach-α = 0.88, 95%-CI = 0.86–0.89, *N* = 454 in Pakistan] was used to measure severity of depressive symptoms. The Generalized Anxiety Disorder-7 [GAD-7 (31); Bangladesh: Cronbach-α = 0.85, 95%-CI = 0.83–0.87, *N* = 845; Pakistan: Cronbach-α = 0.90, 95%-CI = 0.89–0.92, *N* = 454] was used to measure the severity of anxiety symptoms. Mental wellbeing was measured using the Short Warwick-Edinburgh Mental Well-being Scale [sWEMWBS (32); Bangladesh: Cronbach-α = 0.91, 95%-CI = 0.90–0.92, *N* = 845; Pakistan: Cronbach-α = 0.82, 95%-CI = 0.80–0.85, *N* = 454]. All three measures were translated by Psychiatrists at NIMH and IOP who were fluent both in the local language (Bengali/Urdu) and English in the subject area via forward and backward translation, similarity review, and final versions were pilot-tested with *n* = 50 people with SMI in Bangladesh and *n* = 15 people with SMI in Pakistan. Participants were also asked to identify their three top worries related to the pandemic.

#### Health-Related Quality of Life

We used the Urdu and Bangla validated versions of EQ-5D-5L provided by the EuroQol Group (33) including the visual analog scale (EQ-5D-VAS). *Livelihood, financial and housing impact:* We asked about changes in these due to the pandemic.

#### Data From IMPACT Survey

Demographic data (gender, age, education, employment status, monthly income, and marital status) were taken from the IMPACT survey (25).

### Statistical Analyses

Quantitative data were summarized using descriptive statistics, both overall and by site.

Two outcome variables were constructed for the main analysis, (i) knowledge of COVID-19 and (ii) practice relating to COVID-19. We calculated the total number of correct responses to questions related to “knowledge” of COVID-19 symptoms and prevention measures; and the total number of practices reported for controlling COVID-19 [reporting Cronbach-α as an estimate of this scores reliability including 95% confidence intervals based on *b* = 500 bootstrap samples (34)]. Since we were interested in identifying individuals with relatively “poor” knowledge or practice relating to the pandemic, we dichotomised these scores at their first quartile.

For each country's data, one regression analysis for knowledge and one for practice were conducted to explore the potential prognostic value of participants' characteristics. The predictor variables used in the analyses included both demographic data [gender, age, employment status, average monthly income (log transformed), level of education, and marital status] and health data (MINI diagnosis; PHQ-9, GAD-7, and sWEMWBS scores). Additionally, we included the continuous EQ-5D-VAS score, and specific items from the EQ-5D-5L as binary variables, relating to problems with pain/discomfort, mobility, self-care, and usual activities (all dichotomised as “no problems” vs. “any problems;” the anxiety/depression dimension was excluded due to inclusion of the PHQ-9, GAD-7 and sWEMWBS). We also included sources of information on the pandemic (television, radio, internet websites, and social media). Additionally, we included the date of interview, indexed at the time of the first interview, to account for potential changes in knowledge or practice as the pandemic evolved. When predicting practice relating to the pandemic, we also included knowledge of prevention measures as an independent variable.

Participants were drawn from the previous IMPACT survey; therefore we explored potential systematic drop-out by country (see Appendix). As no strong predictors were identified, no statistical correction was performed. Similarly, no sensitivity analyses for missing data were performed as only *n* = 3 (0.2%) participants were excluded when using listwise deletion. We used regularized logistic regression models [relaxed least absolute shrinkage and selection operator; (35, 36)] to identify variables with robust predictive value based on their cross-validation deviance. In this regression approach the coefficients of a regression model are shrunk against zero based on the cross-validation performance of that model. The shrinkage parameter for our reported models was determined using 5-fold cross-validation and we report the coefficient estimates for the regression model at one standard error above (i.e., in the direction of a more stringent penalty) the lowest cross-validation deviance. Instead of basing the results on a single run of the variable selection, we explored the variability of our results in 1,000 bootstrap samples repeating this procedure. We report the average estimate and its 95% confidence interval across the bootstrap samples for each variable (37); and we also calculated the number of times each variable was included with a non-zero coefficient in our 1,000 bootstrap samples. Analyses were performed in R (R Core 24) (38) with the package glmnet (36).

## Results

**Figure 1:**
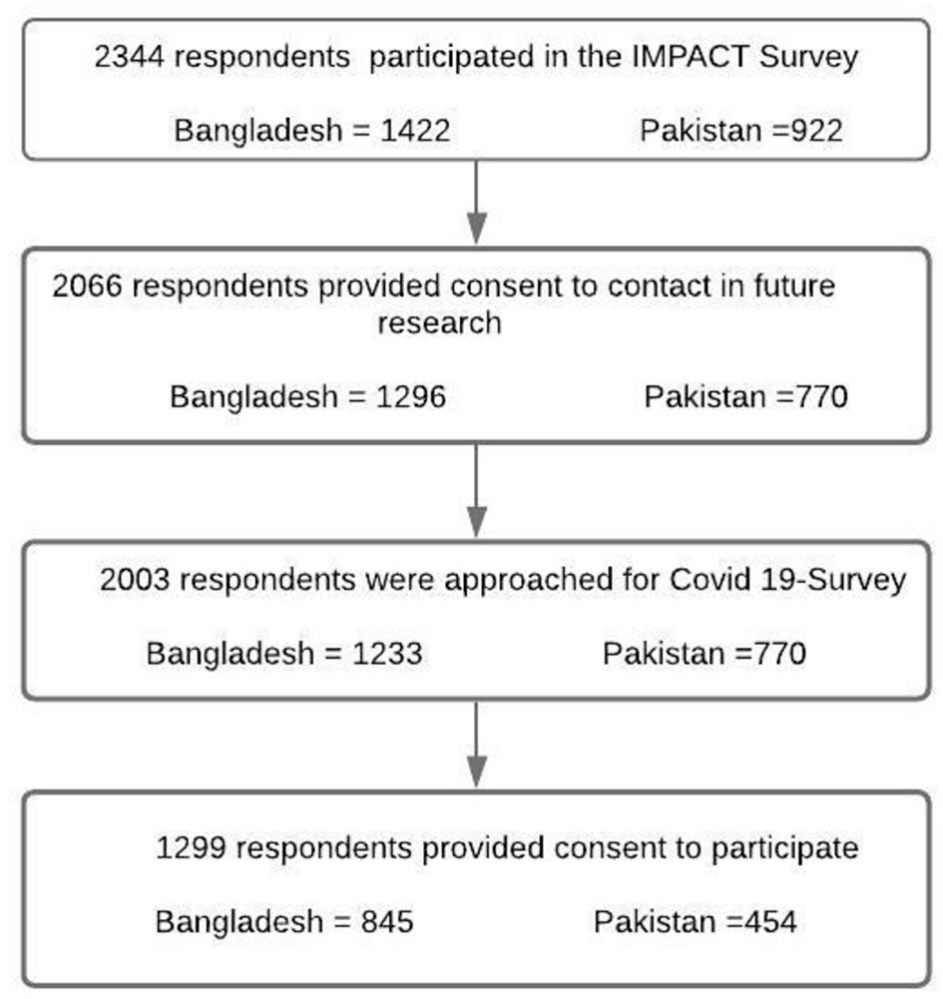
Participant flow chart across the two surveys.

Before the pandemic, participants had been recruited (1,422 in Bangladesh; 922 in Pakistan). Of these, 87% in Bangladesh and 84% in Pakistan had consented to future contact; 59% and 49% of respondents from Bangladesh and Pakistan agreed to participant in this survey, respectively (see Figure 1 for details).

### Participant Characteristics

Table 1 displays participant demographics. Apart from a similar gender-split in both samples (overall 37% were women), the sample distributions differed for most variables. More respondents in Pakistan lived in urban areas (Pakistan 60%; Bangladesh 31%); and in Pakistan respondents were more likely to have had no formal education or secondary education compared with Bangladesh. No participant in Pakistan reported experience of COVID-19 symptoms, while 9.6% in Bangladesh reported such symptoms, but very few were tested for COVID-19 and no positive test results were reported.

**Table 1:** General characteristics of the sample at baseline.

	**Bangladesh (*N* = 845)**	**Pakistan (*N* = 454)**	**Overall (*N* = 1,299)**
	***n* (%) [95% C.I.]**	***n* (%) [95% C.I.]**	***n* (%) [95% C.I.]**
Gender (Female)	321 (38.0) [34.8–41.3]	158 (34.8) [30.5–39.3]	479 (36.9) [34.3–39.5]
Living in Urban area	263 (31.1) [28.1–34.3]	274 (60.4) [55.8–64.8]	537 (41.3) [38.8–43.9]
Age (years)^*^	31.8 (10.7) [31.1–32.6]	38.7 (12.4) [37.5–39.8]	34.2 (11.8) [33.6–34.8]
**Age groups**			
<25	236 (27.9) [25.0–31.1]	50 (11.0) [8.4–14.2]	286 (22.0) [19.9–24.3]
25–34 years	314 (37.2) [34.0–40.5]	126 (27.8) [23.8–32.1]	440 (33.9) [31.4–36.5]
35–44 years	171 (20.2) [17.7–23.1]	141 (31.1) [27.0–35.5]	312 (24.0) [21.8–26.4]
45–54 years	80 (9.5) [7.7–11.6]	83 (18.3) [15.0–22.1]	163 (12.5) [10.9–14.5]
55 or more years	44 (5.2) [3.9–6.9]	54 (11.9) [9.2–15.2]	98 (7.5) [6.2–9.1]
**Education**			
No formal education	78 (9.2) [7.5–11.4]	74 (16.3) [13.2–20.0]	152 (11.7) [10.1–13.6]
Primary	467 (55.3) [51.9–58.6]	76 (16.7) [13.6–20.5]	543 (41.8) [39.3–44.3]
Secondary/higher	300 (35.5) [32.3–38.8]	304 (67.0) [62.5–71.1]	604 (46.5) [43.9–49.1]
Monthly income in last year^*^	232.3 (433.2) [203.1–261.6]	213.4 (218.1) [193.2–233.5]	225.7 (372.7) [205.4–246.1]
**Roof materials of household**			
Raw (Bamboo/Palm leaf/ Straw/Hessian)	5 (0.6) [0.2–1.4]	37 (8.1) [6.0–11.1]	42 (3.2) [2.4–4.3]
Tin/Tally/Similar materials	590 (69.8) [66.6–72.8]	60 (13.2) [10.4–16.7]	650 (50.0) [47.7–52.3]
Cement/Concrete	250 (29.6) [26.6–32.8]	352 (77.5) [73.5–81.1]	602 (46.3) [43.9–48.8]
Electricity in the household	825 (97.6) [96.4–98.5]	451 (99.3) [98.0–99.8]	1276 (98.2) [97.4–98.8]
Flush toilet in the household	506 (59.9) [56.5–63.1]	442 (97.4) [95.4–98.5]	948 (73.0) [70.7–75.1]
Can acces the Internet from home	241 (28.5) [25.6–31.7]	185 (40.7) [36.3–45.3]	426 (32.8) [30.3–35.4]
**Occupation**			
Government employee	12 (1.4) [0.8–2.5]	28 (6.2) [4.3–8.8]	40 (3.1) [2.3–4.2]
Non-government employee	91 (10.8) [8.8–13.0]	132 (29.1) [25.1–33.4]	223 (17.2) [15.3–19.3]
Self-employed	154 (18.2) [15.8–21.0]	31 (6.8) [4.8–9.6]	185 (14.2) [12.5–16.2]
Non-paid	10 (1.2) [0.6–2.2]	6 (1.3) [0.6–2.9]	16 (1.2) [0.8–2.0]
Student	81 (9.6) [7.8–11.8]	10 (2.2) [1.2–4.0]	91 (7.0) [5.7–8.5]
Homemaker	199 (23.6) [20.8–26.5]	130 (28.6) [24.7–33.0]	329 (25.3) [23.0–27.8]
Retired	0 (0.0)	8 (1.8) [0.9–3.5]	8 (0.6) [0.3–1.2]
Unemployed (able to work)	161 (19.1) [16.5–21.8]	95 (20.9) [17.4–24.9]	256 (19.7) [17.6–22.0]
Unemployed (unable to work)	137 (16.2) [13.9–18.9]	14 (3.1) [1.8–5.1]	151 (11.6) [10.0–13.4]
Currently married/living with partner	466 (55.1) [51.8–58.5]	250 (55.1) [50.5–59.6]	716 (55.1) [52.4–57.8]
Currently married (Male)	253 (48.3) [44.0–52–6]	157 (53.0) [47.3–58.7]	410 (50.0) [46.6–53.4]
Currently married (Female)	213 (66.4) [61.0]−71.3]	93 (58.9) [51.0–66.3]	306 (63.9) [59.5–68.1]
**Severe mental illness diagnosis**			
Bipolar disorder	279 (33.0) [29.9–36.3]	200 (44.1) [39.5–48.7]	479 (36.9) [34.3–39.5]
Psychosis	522 (61.8) [58.4–65.0]	51 (11.2) [8.6–14.5]	573 (44.1) [41.8–46.5]
Major depressive disorder with psychotic features	44 (5.2) [3.9–6.9]	203 (44.7) [40.2–49.3]	247 (19.0) [17.2–21.0]
**Type or setting of patient**			
Inpatient	179 (21.2) [18.6–24.1]	38 (8.4) [6.1–11.3]	217 (16.7) [14.8–18.8]
Outpatient	666 (78.8) [75.9–81.4]	416 (91.6) [88.7–93.9]	1,082 (83.3) [81.2–85.2]

* *Values presented as mean (S.D.) [95% C.I.]*.

### Mental Health and Health-Related Quality of Life

Thirty-seven percent of participants had bipolar disorder, 44% non-affective psychosis (higher in Bangladesh), and 19% major depressive disorder with psychotic features (higher in Pakistan; see Table 2 for details). Thirty percent reported moderate or severe depressive symptoms (PHQ-9) and 18% reported moderate or severe anxiety symptoms (GAD-7). Participants reported problems with mobility (30%), self-care (24%), usual activities (38%), pain/discomfort (46%), and anxiety/depression (58%) on the EQ-5D-5L. Mean EQ-5D-VAS score was 70.1 (SD = 20.1).

**Table 2:** Mental health, wellbeing, health-related quality of life, and top worries of people with SMI in South Asia during the COVID-19 epidemic.

	**Bangladesh (*N* = 845)**	**Pakistan (*N* = 454)**	**Overall (*N* = 1,299)**
	***n* (%) [95% C.I.]**	***n* (%) [95% C.I.]**	***n* (%) [95% C.I.]**
**Mental health “symptom severity”**			
Severity of depressive symptoms			
PHQ-9 score^*^	6.9 (5.5) [6.5–7.3]	7.3 (6.3) [6.8–7.9]	7.1 (5.8) [6.8–7.4]
None- or minimal (0–4)	339 (40.1) [36.9–43.5]	177 (39.0) [34.6–43.6]	516 (39.7) [37.1–42.4]
Mild (5–9)	255 (30.2) [27.2–33.4]	138 (30.4) [26.3–34.8]	393 (30.3) [27.8–32.8]
Moderate (10–14)	163 (19.3) [16.8–22.1]	69 (15.2) [12.2–18.8]	232 (17.9) [15.9–20.0]
Moderately severe (15–19)	77 (9.1) [7.3–11.3]	42 (9.3) [6.9–12.3]	119 (9.2) [7.7–10.9]
Severe (≥20)	11 (1.3) [0.7–2.3]	28 (6.2) [4.3–8.8]	39 (3.0) [2.2–4.1]
**Severity of anxiety symptoms**			
GAD7 score^*^	4.5 (4.3) [4.3–4.8]	5.5 (5.4) [5.0–6.0]	4.9 (4.7) [4.6–5.1]
None- or minimal (0–4)	481 (56.9) [53.6–60.2]	250 (55.1) [50.5–59.6]	731 (56.3) [53.6–59.0]
Mild (5–9)	230 (27.2) [24.3–30.3]	100 (22.0) [18.4–26.1]	330 (25.4) [23.1–27.8]
Moderate (10–14)	118 (14.0) [11.8–16.5]	59 (13.0) [10.2–16.4]	177 (13.6) [11.9–15.6]
Severe (15–21)	16 (1.9) [1.2–3.1]	45 (9.9) [7.5–13.0]	61 (4.7) [3.7–6.0]
**Measure of mental wellbeing**			
sWEMWBS Score^*^	22.0 (6.8) [21.6–22.5]	17.4 (5.9) [16.9–18.0]	20.4 (6.9) [20.0–20.8]
Low (<20)	316 (37.4) [34.2–40.7]	330 (72.7) [68.4–76.6]	646 (49.7) [47.2–52.3]
Moderate (20–27)	318 (37.6) [34.4–41.0]	77 (17.0) [13.8–20.7]	395 (30.4) [28.0–32.9]
High (>27)	211 (25.0) [22.2–28.0]	47 (10.4) [7.9–13.5]	258 (19.9) [17.8–22.1]
**Health related quality of life**			
Visual analog scale^*^	69.8 (19.1) [68.5–71.1]	70.8 (21.8) [68.8–72.8]	70.1(20.1) [69.0–71.2]
Mobility	194 (23.0) [20.2–25.9]	189 (41.6) [37.2–46.2]	383 (29.5) [27.1–32.0]
Self–care	187 (22.1) [19.5–25.1]	126 (27.8) [23.8–32.1]	313 (24.1) [21.8–26.5]
Usual activities	329 (38.9) [35.7–42.3]	163 (35.9) [31.6–40.4]	492 (37.9) [35.3–40.6]
Pain/discomfort	370 (43.8) [40.5–47.2]	228 (50.2) [45.6–54.8]	598 (46.0) [43.3–48.8]
Anxiety/ depression	526 (62.2) [58.9–65.5]	225 (49.6) [45.0–54.2]	751 (57.8) [55.1–60.5]
**Covid-19 pandemic worries**			
Income or earnings	404 (47.8) [44.5–51.2]	214 (47.1) [42.6–51.7]	618 (47.6) [44.9–50.3]
Physical illness	233 (27.6) [24.7–30.7]	62 (13.7) [10.8–17.1]	295 (22.7) [20.5–25.0]
Employment after the coronavirus epidemic	76 (9.0) [7.2–11.1]	118 (26.0) [22.2–30.2]	194 (14.9) [13.1–16.9]

* *Values presented as mean (S.D.) [95% C.I.]*.

### Knowledge, Practice, and Information Sources Related to the COVID-19 Pandemic

Most participants had good knowledge of symptoms and control measures, and generally reported good compliance with COVID-19 prevention measures (Table 3). Of the correct potential symptoms, the least well-known was a sore throat/hoarse voice (60% in Pakistan); the least well-known correct practice was around self-isolation (70% in Pakistan); and washing hands before leaving home, not meeting others including friends and family, and using hand sanitizing gel were less practiced (around or below 50%). Participants also reported better practices (Table 4) and at the time of the survey, 8% were in self isolation, with 2% of the respondents in household isolation. The main sources of information about COVID-19 were through television (80%) followed by family and friends (77%), and religious leaders (38%; Table 5).

**Table 3:** Knowledge relevant to the COVID-19 epidemic.

	**Bangladesh (*N* = 845)**	**Pakistan (*N* = 454)**	**Overall (*N* = 1,299)**
	***n* (%) [95% C.I.]**	***n* (%) [95% C.I.]**	***n* (%) [95% C.I.]**
**Knowledge about Covid-19 symptoms (proportion of participants that responded yes)**			
Individuals who believe that the following is a symptom of Covid-19			
A dry cough	764 (90.4) [88.2–92.2]	377 (83.0) [79.3–86.2]	1,141 (87.8) [86.0–89.5]
Breathing difficulties/shortness of breath	753 (89.1) [86.8–91.0]	367 (80.8) [76.9–84.2]	1,120 (86.2) [84.2–88.0]
Fever	789 (93.4) [91.5–94.9]	402 (88.5) [85.3–91.2]	1,191 (91.7) [90.1–93.1]
Sore throat/hoarse voice	737 (87.2) [84.8–89.3]	273 (60.1) [55.5–64.5]	1,010 (77.8) [75.5–79.8]
Bleeding (internal or external)^‡^	137 (16.2) [13.9–18.9]	31 (6.8) [4.8–9.6]	168 (12.9) [11.2–14.9]
Rash^‡^	165 (19.5) [17.0–22.3]	44 (9.7) [7.3–12.8]	209 (16.1) [14.2–18.2]
Vomiting^‡^	333 (39.4) [36.2–42.8]	56 (12.3) [9.6–15.7]	389 (29.9) [27.6–32.4]
Median number of correct responses (IQR)	5 (2)	5 (3)	5 (2)
**Knowledge about Covid-19 prevention (proportion of participants that responded yes)**			
Coronavirus is contagious (can be caught from other people)	765 (90.5) [88.4–92.3]	390 (85.9) [82.4–88.8]	1,155 (88.9) [87.1–90.5]
Maintaining at least 2 m (6 feet) distance between yourself and another person, may help reduce your risk of infection	748 (88.5) [86.2–90.5]	366 (80.6) [76.7–84.0]	1,114 (85.8) [83.8–87.5]
Washing your hands with soap and water may help reduce your risk of infection	769 (91.0) [88.9–92.8]	344 (75.8) [71.6–79.5]	1,113 (85.7) [83.7–87.4]
If someone in your household has symptoms of coronavirus, you should self-isolate (not leave the house) for 14 days	735 (87) [84.5–89.1]	319 (70.3) [65.9–74.3]	1,054 (81.1) [79.0–83.1]
Shaking hands with people may increase your risk of getting infected with coronavirus	732 (86.6) [84.2–88.8]	346 (76.2) [72.1–79.9]	1,078 (83.0) [80.9–84.9]
Antibiotics are effective in preventing and treating coronavirus^‡^	228 (27.0) [24.1–30.1]	120 (26.4) [22.6–30.7]	348 (26.8) [24.4–29.3]
Regularly rinsing your nose with saline will help reduce the risk of coronavirus^‡^	328 (38.8) [35.6–42.2]	295 (65.0) [60.5–69.2]	623 (48.0) [45.3–50.6]
Exercise inside will increase your risk of infection^‡^	176 (20.8) [18.2–23.7]	80 (17.6) [14.4–21.4]	256 (19.7) [17.6–22.0]
Median number of correct responses (IQR)	5 (0)	5 (1)	5 (1)

‡ *Symptoms and prevention related knowledge that were counted as incorrect responses*.

**Table 4:** Attitudes and practice relevant to the COVID-19 epidemic.

	**Bangladesh (*N* = 845)**	**Pakistan (*N* = 454)**	**Overall (*N* = 1,299)**
	***n* (%) [95% C.I.]**	***n* (%) [95% C.I.]**	***n* (%) [95% C.I.]**
**Practice regarding covid-19**			
I go out as little as I possibly can	723 (85.6) [83.0–87.8]	373 (82.2) [78.4–85.4]	1096 (84.4) [82.3–86.2]
When I go out, I stay at least 2 m (6 feet) away from other people at all times.	755 (89.3) [87.1–91.3]	349 (76.9) [72.8–80.5]	1104 (85.0) [83.0–86.8]
When I go out, I wear a face mask.	783 (92.7) [90.7–94.2]	378 (83.3) [79.5–86.4]	1161 (89.4) [87.6–90.9]
I always wash my hands immediately before I leave home.	459 (54.3) [50.9–57.7]	287 (63.2) [58.7–67.5]	746 (57.4) [54.7–60.1]
I always wash my hands as soon as I get home.	770 (91.1) [89.0–92.9]	368 (81.1) [77.2–84.4]	1138 (87.6) [85.7–89.3]
I do not meet others, even friends or family, who don't live in my home.	443 (52.4) [49–55.8]	208 (45.8) [41.3–50.4]	651 (50.1) [47.4–52.8]
I use disinfectants to wash surfaces in my home more frequently than before the coronavirus epidemic.	539 (63.8) [60.5–67.0]	206 (45.5) [40.8–50.0]	745 (57.4) [54.7–60.0]
I wash my hands with soap and water more often than before the coronavirus epidemic.	754 (89.2) [87.0–91.2]	276 (60.8) [56.2–65.2]	1030 (79.3) [77.1–81.3]
I use hand sanitizing gel if soap and water are not available.	222 (26.3) [23.4–29.4]	171 (37.7) [33.3–42.2]	393 (30.3) [27.8–32.8]
**Self-isolation practices**			
I am self-isolating now (that is, not leaving the house at all, even for shopping).	83 (9.8) [8.0–12.0]	14 (3.1) [1.8–5.1]	97 (7.5) [6.2–9.0]
Duration of self-isolation^**^	55.9 (23.5) [50.8–61.0]^*^	36.8 (28.5) [17.9–55.7]^*^	54.0 (24.5) [49.1–59.0]^*^
My household is self-isolating now	21 (2.5) [1.6–3.8]	8 (1.8) [0.9–3.5]	29 (2.2) [1.6–3.2]
Duration of self-isolation household^**^	32.2 (11.8) [26.9–37.5]	20.8 (10.2) [12.3–29.4]	29.7 (12.3) [25.1–34.2]

* *Values presented as mean (S.D.) [95% C.I.]*.

** *Quarantine in days*.

**Table 5:** Source of information relevant to the COVID-19 epidemic.

	**Bangladesh (*N* = 845)**	**Pakistan (*N* = 454)**	**Overall (*N* = 1,299)**
	***n* (%) [95% C.I.]**	***n* (%) [95% C.I.]**	***n* (%) [95% C.I.]**
**Sources of information**			
Family and friends	731 (86.5) [84–88.7]	272 (59.9) [55.3–64.3]	1,003 (77.2) [75–79.3]
Government agencies	152 (18) [15.5–20.7]	179 (39.4) [35–44]	331 (25.5) [23.2–27.9]
My doctor	61 (7.2) [5.7–9.2]	178 (39.2) [34.8–43.8]	239 (18.4) [16.5–20.4]
Other health professionals	62 (7.3) [5.8–9.3]	134 (29.5) [25.5–33.9]	196 (15.1) [13.3–17]
Social media (Facebook, WhatsApp)	176 (20.8) [18.2–23.7]	87 (19.2) [15.8–23.1]	263 (20.2) [18.1–22.5]
Internet websites	150 (17.8) [15.3–20.5]	54 (11.9) [9.2–15.2]	204 (15.7) [13.8–17.8]
Radio	38 (4.5) [3.3–6.1]	39 (8.6) [6.3–11.5]	77 (5.9) [4.8–7.3]
Television	678 (80.2) [77.4–82.8]	357 (78.6) [74.6–82.2]	1,035 (79.7) [77.4–81.8]
Newspapers	124 (14.7) [12.4–17.2]	71 (15.6) [12.6–19.3]	195 (15) [13.2–17.1]
Religious leaders	331 (39.2) [35.9–42.5]	168 (37) [32.7–41.6]	499 (38.4) [35.8–41.1]

### Change in Family Support, Finances, Housing, and Food Security

With respect to family support and housing (Table 6), participants reported three (SD = 5.00) people within walking distance, who could be counted upon in time of need. Thirty-three percent of the main earners of the family were not currently working and 60% were worried about the job/business security of the main earner. Thirty percent reported serious difficulties and 67% considered their financial stability was worse than pre-pandemic. Twelve percent had received government aid in the form of emergency funds during the pandemic. Thirty-nine percent indicated that their household was unable to eat preferred food, and 35% exhibited worries about insufficient food. When asked about their top three worries about the impact of the pandemic, participants mentioned income/earnings (48%), physical illness (23%), and employment (15%).

**Table 6:** Family support and isolation, livelihood, financial, and housing related issues during the COVID-19 epidemic.

	**Bangladesh (*N* = 845)**	**Pakistan (*N* = 454)**	**Overall (*N* = 1,299)**
	***n* (%) [95% C.I.]**	***n* (%) [95% C.I.]**	***n* (%) [95% C.I.]**
**Family support and isolation**			
No of people in household^*^	5.4 (2.2) [5.3–5.6]	6.8 (3.4) [6.4–7.1]	5.9 (2.8) [5.7–6.0]
Living alone (ie only 1 member in household)^*^	0 (0)	6 (1.3) [0.6–2.9]	6 (0.5) [0.2–1.0]
No of people (friends/family /neighbors) who can usually be counted on, in the time of need^*^	8.5 (19.4) [7.2–9.8]	2.9 (3.6) [2.6–3.3]	6.6 (16.0) [5.7–7.4]
No of people living within walking distance (today and past week) who could be counted on, in time of need^*^	3.1 (6.1) [2.7–3.5]	2.1 (3.0) [1.9–2.4]	2.8 (5.3) [2.5–3.1]
**Livelihood**			
**Main earner in the household**			
*SMI patient*	167 (19.8) [17.2–22.6]	149 (32.8) [28.6–37.3]	316 (24.3) [22.1–26.7]
*Other family member*	678 (80.2) [77.4–82.8]	305 (67.2) [62.7–71.4]	983 (75.7) [73.3–77.9]
Main earner of the household currently employed/running business	544 (64.4) [61.1–67.5]	287 (63.2) [58.7–67.5]	831 (64.0) [61.3–66.5]
Main earners who are employed/running business but not currently working	235 (43.2) [39.1–47.4]	40 (13.9) [10.4–18.5]	275 (33.1) [30.1–36.2]
**Mode of work of the main earners who are currently working:**			
*Working from home*	55 (17.8) [13.9–22.5]	24 (9.7) [6.6–14.1]	79 (14.2) [11.6–17.4]
*Go to office /outside to work*	254 (82.2) [77.5–86.1]	223 (90.3) [85.9–93.4]	477 (85.8) [82.6–88.4]
Worried about the job/business security of the main earner	395 (72.6) [68.7–76.2]	103 (35.9) [30.5–41.6]	498 (59.9) [56.8–63.0]
**Financial and housing related issues**			
**Management of finances in the household**			
*Doing alright*	216 (25.6) [22.7–28.6]	47 (10.6) [8.1–13.9]	263 (20.4) [18.3–22.7]
*Just about getting by*	341 (40.4) [37.1–43.7]	292 (66.1) [61.5–70.3]	633 (49.2) [46.5–51.8]
*Finding it very difficult*	281 (33.3) [30.2–36.5]	101 (22.9) [19.2–27.0]	382 (29.7) [27.3–32.2]
*Do not wish to answer*	7 (0.8) [0.4–1.7]	2 (0.5) [0.1–1.8]	9 (0.7) [0.4–1.3]
**Financial stablity compared to 3 months ago**			
*Worse off*	637 (75.4) [72.4–78.2]	235 (51.8) [47.2–56.3]	872 (67.1) [64.6–69.6]
Received emergency funds from the government during the coronavirus crisis	91 (10.8) [8.8–13]	64 (14.1) [11.2–17.6]	155 (11.9) [10.3–13.8]
Worried about paying house rent /house loan	176 (20.8) [18.2–23.7]	96 (21.1) [17.6–25.2]	272 (20.9) [18.8–23.2]
Worried about getting evicted or losing your home	103 (12.2) [10.1–14.6]	77 (17) [13.8–20.7]	180 (13.9) [12.1–15.8]
Household updated with all bills	230 (27.2) [24.3–30.3]	198 (43.6) [39.1–48.2]	428 (32.9) [30.5–35.5]
**Food security**			
Worried that household would not have enough food	327 (38.7) [35.5–42]	123 (27.1) [23.2–31.4]	450 (34.6) [32.1–37.3]
Household member not able to eat preferred food due to lack of resources	415 (49.1) [45.7–52.5]	92 (20.3) [16.8–24.2]	507 (39) [36.5–41.6]
Household members had smaller meal due—food was not enough	234 (27.7) [24.8–30.8]	N/A	234 (27.7) [24.8–30.8]
No food at household—lack of resources to get food	153 (18.1) [15.6–20.9]	N/A	153 (18.1) [15.6–20.9]
Slept late at night hungry—not enough food	91 (10.8) [8.8–13]	N/A	91 (10.8) [8.8–13]

* *Values presented as mean (S.D.) [95% C.I.]*.

*N/A Responses to these three questions were not collected due to their potentially sensitive nature*.

### The Association Between Demographic Variables, Mental Health, and Knowledge and Response to the Pandemic

**Figure 2:**
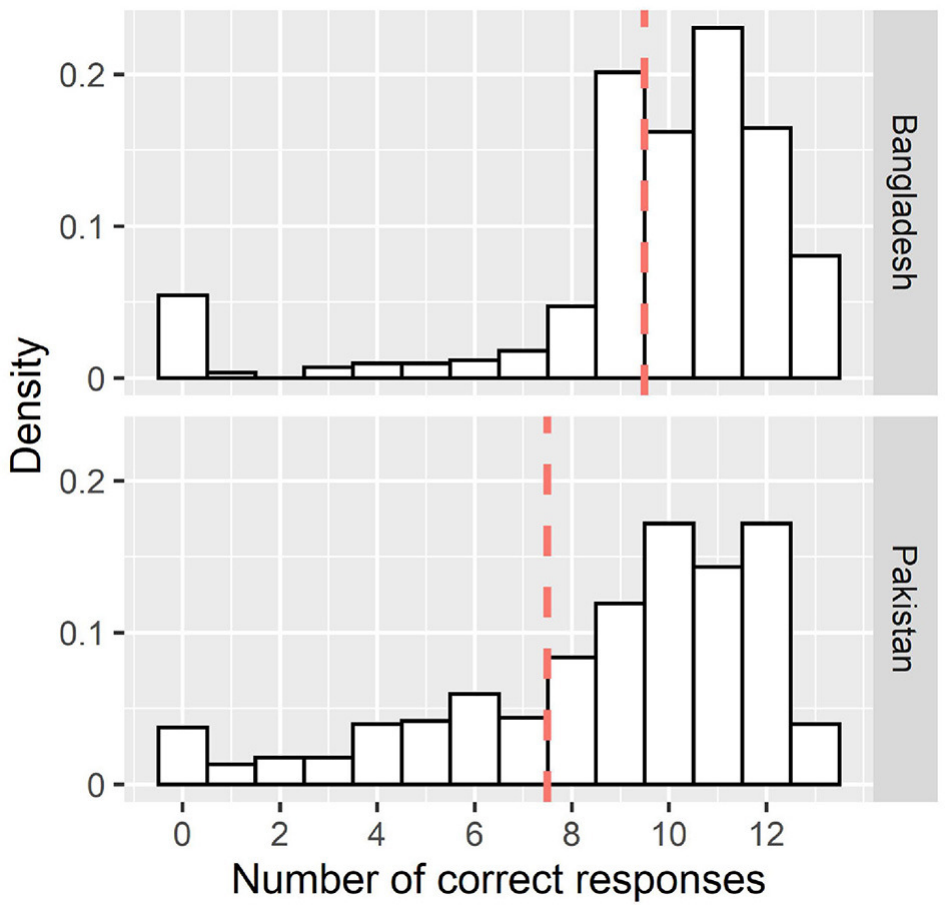
Distribution of the number of correct responses to the 13 questions relating to symptoms of COVID-19. The dashed line represents the cut off to determine “poor” Knowledge (lower 25% of the distribution).

In Bangladesh, the mean number of correct responses to the 13 questions relating to COVID-19 symptoms (seven questions) and prevention measures (6 questions) was 9.70 (SD = 3.00; Cronbach-α = 0.87, 95%CI = 0.84–0.89, *N* = 845) and scores of nine or fewer correct responses were classified as relatively poor knowledge (*n* = 306; 36%; Figure 2). In the text, we focus on variables that were consistently included in the regularized regression models (80%/>800 times; full results can be found in Table 7). Being female was a prognostic factor (OR = 1.44) of poor knowledge. Higher EQ-5D-VAS score (OR = 0.97) and receiving information from the television (OR = 0.41) were indicative of reduced risk of poor knowledge (Table 7). In Pakistan, the mean number of correct responses was 8.7 (SD = 3.23; Cronbach-α = 0.84, 95%CI = 0.82–0.86, *N* = 454). Scores indicating seven or fewer correct responses were classified as relatively poor knowledge (*n* = 123; 27%). Being female (OR = 0.75) and receiving information from television (OR = 0.51) were indicative of reduced risk of poor knowledge. While reliably identified in replications, the confidence intervals for the coefficient estimates of gender included values close to OR = 1.00 (after rounding equal to 1.00) in both countries, as did the interval for TV as the main information source in the sample from Pakistan. Especially since these are dichotomous variables, the effect size range includes values that may be negligible from a practical point of view.

**Table 7:** Results of the regularized logistic regression models (*N* = 845 Bangladesh; *N* = 451 Pakistan) predicting comparatively poor knowledge and limited prevention practice.

**Variable**	**“Poor”**		**“Poor”**		**“Poor”**		**“Poor”**	
	**knowledge—Bangladesh**		**knowledge—Pakistan**		**practice—Bangladesh**		**practice—Pakistan**	
	**OR (95% CI)**	**Freq**.	**OR (95% CI)**	**Freq**.	**OR (95% CI)**	**Freq**.	**OR (95% CI)**	**Freq**.
Inpatient	0.97 (0.78–1.00)	215	1.09 (1.00–1.81)	239	1.01 (0.98–1.10)	113	1.00 (0.93–1.00)	50
Interview date	1.00 (1.00–1.01)	169	1.01 (1.00–1.04)	544	1.00 (1.00–1.02)	377	1.01 (1.00–1.04)	684
Monthly income (USD)	1.00 (1.00–1.01)	50	0.99 (0.90–1.00)	113	0.99 (0.85–1.00)	219	0.97 (0.80–1.00)	341
Age	1.00 (1.00–1.00)	34	1.00 (1.00–1.00)	57	1.00 (0.99–1.00)	417	1.00 (1.00–1.00)	37
Unemployed	1.05 (1.00–1.39)	305	1.00 (0.95–1.07)	79	1.00 (0.95–1.08)	101	1.00 (1.00–1.08)	56
Homemaker	1.05 (1.00–1.43)	243	1.00 (1.00–1.00)	19	0.99 (0.88–1.00)	75	0.94 (0.57–1.00)	272
Student	0.97 (0.75–1.00)	204	0.99 (0.74–1.00)	65	1.05 (1.00–1.44)	220	0.90 (0.24–1.00)	233
MINI diagnosis: major depressive disorder	1.01 (1.00–1.08)	54	0.97 (0.74–1.00)	208	0.86 (0.45–1.00)	525	1.00 (1.00–1.00)	32
MINI diagnosis: bipolar disorder with psychotic feature	1.01 (1.00–1.10)	76	0.99 (0.86–1.00)	61	0.95 (0.75–1.00)	392	1.00 (1.00–1.00)	38
Primary education	1.07 (1.00–1.42)	398	1.07 (1.00–1.55)	257	0.99 (0.90–1.00)	116	1.00 (1.00–1.01)	36
No formal education	1.05 (1.00–1.55)	191	1.02 (1.00–1.31)	121	1.01 (1.00–1.22)	115	1.02 (1.00–1.27)	91
Never married	0.98 (0.82–1.00)	162	1.06 (1.00–1.43)	265	1.00 (1.00–1.02)	58	1.06 (1.00–1.53)	240
Divorced/separated/widowed	1.07 (1.00–1.56)	270	1.08 (1.00–1.71)	220	1.00 (0.89–1.06)	89	1.02 (1.00–1.32)	79
Living urban	1.00 (1.00–1.00)	34	0.99 (0.90–1.00)	77	0.94 (0.69–1.00)	471	0.98 (0.78–1.00)	166
Home internet access	0.99 (0.91–1.00)	74	1.17 (1.00–1.73)	589	0.94 (0.69–1.00)	476	1.00 (1.00–1.00)	30
Female	1.44 (1.00–2.04)	963	0.75 (0.41–1.00)	829	0.49 (0.30–0.76)	999	0.84 (0.46–1.00)	603
SWEMWBS score	0.99 (0.97–1.00)	433	0.99 (0.95–1.00)	629	1.00 (0.98–1.00)	334	1.00 (1.00–1.00)	19
PHQ-9 score	1.00 (1.00–1.00)	14	1.00 (1.00–1.03)	306	1.00 (1.00–1.00)	30	1.00 (1.00–1.00)	19
GAD-7 score	0.99 (0.96–1.00)	303	1.00 (1.00–1.00)	22	0.96 (0.92–1.00)	954	1.00 (0.98–1.00)	117
Accessing information from social media	0.97 (0.77–1.00)	224	0.91 (0.57–1.00)	407	0.96 (0.92–1.00)	985	0.95 (0.64–1.00)	276
Accessing information from the internet	1.01 (1.00–1.14)	56	1.05 (1.00–1.65)	168	0.54 (0.31–0.85)	996	1.03 (1.00–1.39)	104
Accessing information from radio	−1.00 (0.85–1.00)	67	0.91 (0.46–1.00)	340	0.86 (0.40–1.00)	485	1.00 (1.00–1.00)	34
Accessing information from television	0.41 (0.25–0.63)	1,000	0.51 (0.27–1.00)	952	0.67 (0.43–1.00)	968	0.79 (0.44–1.00)	750
Accessing information from a newspaper	1.00 (1.00–1.00)	38	0.7 (0.66–1.00)	193	0.92 (0.61–1.00)	492	1.01 (1.00–1.11)	48
Mobility issues	0.98 (0.78–1.00)	151	1.09 (1.00–1.49)	411	1.00 (1.00–1.05)	54	1.01 (1.00–1.14)	67
Self-care issues	1.01 (1.00–1.04)	33	1.05 (1.00–1.45)	219	1.01 (1.00–1.15)	75	1.01 (1.00–1.14)	67
Difficulty doing usual activities	0.98 (0.81–1.00)	142	0.99 (0.89–1.00)	45	0.96 (0.70–1.00)	292	1.00 (0.98–1.00)	29
Pain/Discomfort	1.00 (1.00–1.02)	35	1.03 (1.00–1.31)	197	1.35 (1.00–1.97)	886	1.25 (1.00–2.03)	691
EQ-5D-VAS score	0.97 (0.96–0.98)	1,000	1.00 (1.00–1.00)	57	0.99 (0.98–1.00)	938	1.00 (1.00–1.00)	61
Poor knowledge of COVID-19 prevention measures					1.16 (1.00–1.66)	665	5.22 (2.72–8.65)	1,000

*OR, Odds ratios (95% confidence intervals) from 1,000 bootstrap model runs (confidence interval determined across all bootstrap estimations incl. those where the coefficient was shrunk to 0); Freq indicating frequency of selection of this coefficient as non-zero (37); inpatient status, reference category “outpatient;” employment status, reference category “currently employed;” MINI diagnosis, reference category “non-affective psychosis;” education level, reference category “secondary/higher education;” marital status, reference category “currently married;” female, reference category male; urban living, reference category “rural”*.

**Figure 3:**
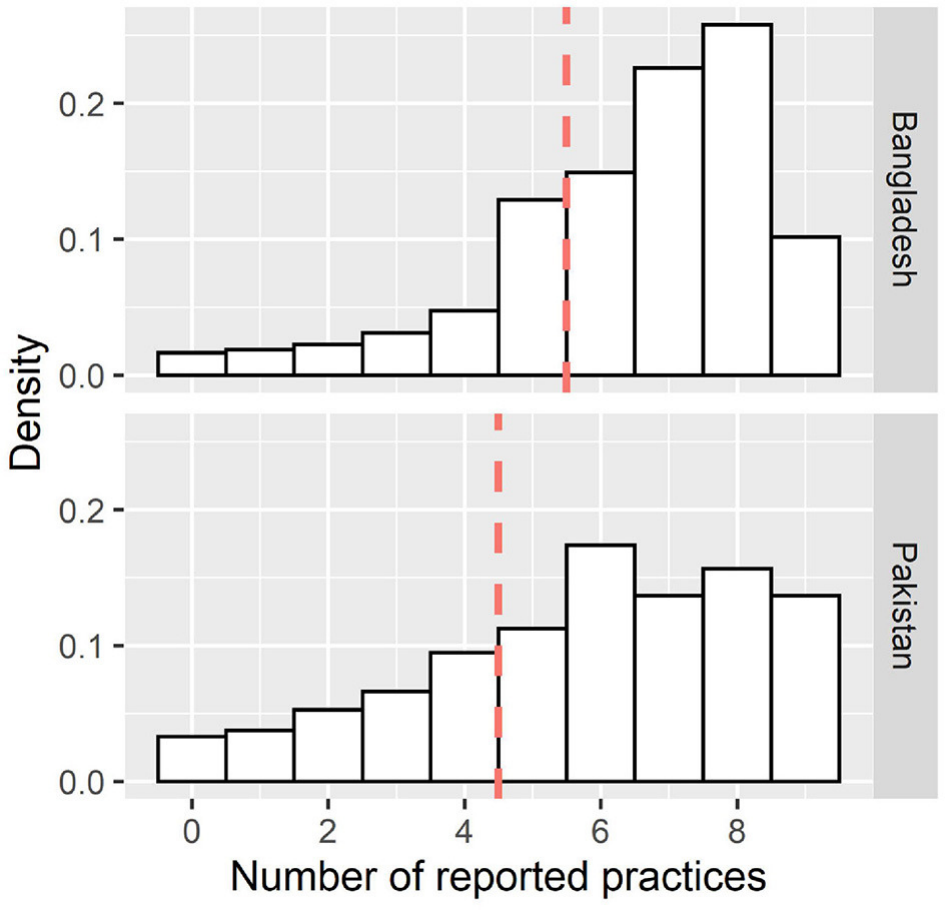
Distribution of the number of reported practices (out of nine) to reduce the spread of COVID-19. The dashed line represents the cut off to determine “poor” practice (lower 25% of the distribution).

The mean number of reported practices related to COVID-19 prevention in Bangladesh was 6.45 (out of nine; SD = 2.00; Cronbach-α = 0.76, 95%CI = 0.71–0.76, *N* = 845; Figure 3), and a score of five or fewer correct responses was classified as relatively limited practice (*n* = 224; 26.5%; Figure 3). When predicting relatively limited practice related to the pandemic in Bangladesh, reporting pain/discomfort was a prognostic factor of poor practice (OR = 1.35). In contrast, being female (OR = 0.49) and receiving information from social media (OR = 0.96), internet (OR = 0.54), and television (OR = 0.67) as well as a higher GAD-7 score (OR = 0.96) or a higher EQ-5D-VAS score (OR = 0.99) reduced the probability of poor practice. Again, the confidence intervals for some of the variables included values very close to OR = 1.00, i.e., the effect size range includes values that may be negligible from a practical point of view. In Pakistan, the mean number of practices reported was 5.76 (SD = 2.45; Cronbach-α = 0.78, 95%CI = 0.78–0.81, *N* = 454) and four or fewer correct responses was classified as relatively limited practice in Pakistan (*n* = 129; 28.4%); only probability of poor knowledge of COVID-19 prevention measures (OR = 5.22) predicted poor practice.

## Discussion

In this paper we sought to describe the pandemic-related experiences of people with SMI, including top concerns, impact on food, financial security and health and knowledge and behaviors relevant to COVID-19, and their associations with demographic and socioeconomic variables and mental health. Despite concerns that people with SMI are less likely to receive information or be in a position to follow public health prevention advice (39), respondents demonstrated good knowledge of coronavirus symptoms and control measures, and reported largely following social distancing and hygiene advice. The general population of Pakistan has also been reported to have good knowledge, optimistic attitude and appropriate practice toward COVID-19 (40), potentially due to the wide penetration of government health messages via mobile phones, television and social and print media (41, 42). When investigating predictors of knowledge of COVID-19 symptoms and practice, receiving information via the television predicted relatively better knowledge. Findings are concordant with studies from Bangladesh (43) and Pakistan (20) reporting social media and news media as the main sources of information (24).

Gender was related to knowledge in both countries. However, unexpectedly, the relationship was reversed across the two sites, with females at higher risk of poor knowledge in Bangladesh, and males at higher risk of poor knowledge in Pakistan. Previous general population studies show mixed results, with males in Bangladesh (44) and females in Pakistan (40) showing better knowledge; while some studies point out that knowledge regarding COVID-19 was similar across age, gender and occupation in Bangladesh (45). Understanding how gender structures knowledge and implementation of hygiene advice during a pandemic in these cultures as well as the population of people living with SMI seems therefore to be an important emerging topic for further research.

Poor knowledge of COVID-19 prevention was a predictor of more limited practice in relation to the pandemic in Pakistan. The literature suggests that knowledge plays an important role in enhancing the practices related to preventive behavior (46); therefore, further improving such knowledge in this population could potentially improve practice relating to the pandemic. In Bangladesh men had worse social distancing and hygiene practices mirroring findings of another study in Bangladesh which indicated that men had less knowledge and poorer hygiene practices compared to women (45).

We observed no relationship between economic factors and knowledge or practices, which is perhaps surprising, as hygiene measures rely on access to cleaning materials (11, 47). However, our measure of income was drawn from the pre-pandemic IMPACT survey, and do not account for changes in income after the start of the pandemic. Finally, it should be noted that many aspects of “good practice” are outside of participants' control such as maintaining social distance and therefore a broader public health approach might be better suited to also benefit the subpopulation of people with SMI in these two countries (11).

### Wider Context

The findings need to be interpreted in the participants' social and historical context. COVID-19 has adversely affected the economy, increasing financial pressures in the general population. Reports on predicted employment indicate an economic recession scenario and spike in poverty in Pakistan (48). Economic recession is also predicted in Bangladesh due to insufficient access to food and social safety net provisions because of COVID-19 (49). Such changes of the economic climate were significantly associated with depression, anxiety and stress among the rehabilitation professional in Bangladesh (50), whereas financial threat and economic hardships were reported as predictors of mental health conditions like depression and anxiety among youth during the pandemic (51). In this context, people with SMI are one of the “hardest hit” populations in the current pandemic, due to their vulnerability to COVID-19 and amplification of challenges such as unemployment, stigma and healthcare (6, 39). Our descriptive results show good social support, but the majority of respondents reported deterioration in finances compared with the pre-pandemic period. Only a minority had received government emergency funds and one third were worried about having insufficient food because of financial limitations. This overall description of the situation suggests that building-in considerations about the financial impact of the pandemic in this disadvantaged population is an important consideration for planning (52).

While we present results from one of the few studies on the experiences related to COVID-19 specifically in people with SMI, one limitation relates to the sample, as the original IMPACT SMI survey was conducted in people attending specialist mental health institutes. Although these institutes see the broad range of mental health conditions, including those that would be typically seen in primary and secondary care in other health systems, their case-mix is likely different from, for example, community samples. Second, only those with capacity who provided consent to contact and were contactable by telephone were included. There are also limitations of remote data collection by telephone (e.g., retaining attention). However, in a situation in which in-person interviews were not possible because of the pandemic, our telephone administered questionnaire provided a means to collect information from the SMI population, who would otherwise likely be excluded by an online survey. Finally, as the data were collected cross-sectionally, we cannot draw causal conclusions, but we were able to identify relevant associations that held robustly when re-sampling from, and cross-validating within, our samples.

## Conclusion

For public health advice to be effective, prevention practices need to be disseminated to all parts of the population including disadvantaged groups such as people living with SMI. It is additionally important to understand the potential concerns and socio-economic constraints for such groups. We found that our respondents had significant concerns about income and finances, employment and physical health and faced financial and food security challenges, with limited support from government emergency funds. Together with worries around healthcare access this raises questions of how to better support this population. With regards to the main analysis, we found that people with SMI in Bangladesh and Pakistan had good knowledge of COVID-19 symptoms and prevention measures, and were following social distancing and hygiene advice. Our analyses emphasize the importance of access to news media for the dissemination of advice and that the uptake of both knowledge and practice is likely gendered, which requires further attention.

## Data Availability Statement

The raw data supporting the conclusions of this article will be made available by the authors, without undue reservation.

## Ethics Statement

The studies involving human participants were reviewed and approved by Department of Health Sciences, University of York, Center for Injury Prevention and Rehabilitation (Bangladesh), Health Ministry Screening Committee and Indian Council of Medical Research (India), and National Bioethics Committee (Pakistan). The patients/participants provided their written informed consent to participate in this study.

## Author Contributions

LP, AC, GZ, and JB: data curation. LP, AC, and JB: formal analysis. RH, PM, AN, KM, DS, NS, and JB: funding acquisition. LP, AC, RH, PM, AN, KM, DS, NS, and JB: methodology. SR, AC, GZ, FA, RH, HK, PM, AN, KM, and NS: project administration. JB and NS: supervision. SR, LP, AC, GZ, NS, and JB: writing—original draft. All authors confirm that they have made substantial contributions to all of the following: (1) the conception and design of the study, acquisition of data, analysis, and interpretation of data, (2) drafting the article or revising it critically for important intellectual content, and (3) final approval of the version to be submitted. All authors conceptualization, investigation, and writing—review and editing.

## Funding

This research was funded by the National Institute for Health Research (NIHR) (17/63/130) using UK aid from the UK Government to support global health research. The views expressed in this publication are those of the author(s) and not necessarily those of the NIHR or the UK government. The funder had no involvement in the study design; in the collection, analysis and interpretation of data; in the writing of the report; or in the decision to submit the article for publication.

## Conflict of Interest

The authors declare that the research was conducted in the absence of any commercial or financial relationships that could be construed as a potential conflict of interest. DS is expert advisor to the NICE centre for guidelines; Board member of the National Collaborating Centre for Mental Health (NCCMH); views are personal and not those of NICE or NCCMH.

## Publisher's Note

All claims expressed in this article are solely those of the authors and do not necessarily represent those of their affiliated organizations, or those of the publisher, the editors and the reviewers. Any product that may be evaluated in this article, or claim that may be made by its manufacturer, is not guaranteed or endorsed by the publisher.
