# Supplementary material for: Knowledge and Response to the COVID-19 Pandemic in People With Severe Mental Illness in Bangladesh and Pakistan: A Cross-Sectional Survey
Source: Front Psychiatry. 2022 Feb 14;13:785059. doi: 10.3389/fpsyt.2022.785059 (PMC8884107; doi:10.3389/fpsyt.2022.785059)
Supplement: Supplementary file 1 [file Data_Sheet_1.pdf]

Supplementary file for "Knowledge and response to the COVID-19 pandemic in people with severe mental illness in Bangladesh and Pakistan: A cross-sectional survey"

Authors:

Sukanya Rajan; Lewis W. Paton; Asiful Haidar Chowdhury; Gerardo A. Zavala; Faiza Aslam; Rumana Huque; Humaira Khalid; Pratima Murthy; Asad T Nizami; Krishna Prasad Muliyala; David Shiers; Najma Siddiqi; Jan R. Boehnke on behalf of the IMPACT research team

Main article DOI: 10.3389/fpsyt.2022.785059, Front. Psychiatry - Public Mental Health

Corresponding Author:

Jan R. Boehnke
j.r.boehnke@dundee.ac.uk
School of Health Sciences, University of Dundee, Dundee, UK
11 Airlie Place, Dundee DD1 4HJ, United Kingdom

Appendix: Propensity to respond to Wave 1

Background

The IMPACT survey is an ongoing survey of health, health risk behaviours and healthcare use in people with severe mental illness (SMI) in Bangladesh, India and Pakistan (ISRCTN registry: 88485933; (1)). Adults aged 18 and over were recruited into the IMPACT survey between July 2019 and March 2020. They were also asked if they consented to be contacted for future surveys. Between May and June 2020, those in the IMPACT survey who consented to being contacted were approached by telephone and asked if they would participate in the first wave of the COVID-19 survey (follow-up survey; ISRCTN registry: 15571919). Systematic non-participation in Wave 1 of the COVID-19 survey would impact on how results from that cross-section of individuals are interpreted. This work thus aims to examine if there are factors contributing to the propensity of IMPACT survey participants to participate in Wave 1 of the COVID-19 survey.

Methods

Data were available for n=2,344 individuals who participated in the original IMPACT survey in Bangladesh (n=1,422) and Pakistan (n=922). Of those, 845 (59.4%) responded to Wave 1 of the COVID-19 survey in Bangladesh, and 454 (49.2%) in Pakistan. Data were also collected in India, but is not being considered in this study (see main paper).

Outcome

The outcome of interest was whether an individual had responded to Wave 1 of the COVID-19 survey.

Predictor variables

All predictor variables were taken from the IMPACT survey. Therefore, where variables are labelled as 'currently', this refers to at the time of the IMPACT survey. We included those variables for which could derive a plausible hypothetical relationship with response. For example, various biological measurements [e.g. calcium levels, cholesterol levels; (1)] were collected in the IMPACT survey, but we did not use them here.

Demographic variables included were gender, age (in years), years of education, formal education received (yes or no), university educated (yes or no), currently married (yes or no), currently employed (yes or no), current student (yes or no), and number of adult housemates. Individual and household economic data included monthly income (in local currency) and binary variables on whether homes had various items, such as electricity, landline phone, mobile phone, tv, a car or a computer. Of these, we included landline phone and mobile phone as predictor variables in this model, since the COVID-19 follow up survey was a telephone-based survey.

We also had access to a variety of mental and physical health variables. These included whether the individual was an inpatient or outpatient at the time of the IMPACT survey, MINI diagnosis (psychotic, major depressive with psychotic, or bipolar), self-reported years with severe mental illness, responses to the Patient Health Questionaire-9 [PHQ-9;(2)]; responses to the Generalised Anxiety Disorder-7 [GAD-7; (3)] and responses to the EQ-5D-VAS (4). We used the total PHQ-9 and GAD-7 scores as continuous variables.

Statistical Analyses

We conducted bivariate logistic regression analyses to identify key characteristics of response to Wave 1 of the COVID-19 survey. Variables statistically significant at the $p<0.05$ level in bivariate analyses were then included in multivariable logistic regression models. Models were built separately in each country.

In order to evaluate the performance of the final models, we calculated the area under the receiver operating characteristic curve (AUC).

Missing data in the predictor variables was imputed using fully conditional specification (30 imputations). The final multivariable regression models were fit on the imputed data. As a sensitivity analysis, coefficients from models fit to the imputed data were compared with those obtained from the non-imputed data.

All data analyses were performed in Stata v.16 (5).

Results

Data were complete for 874 individuals (61.5% of the IMPACT survey) in Bangladesh, with missing data reported for self-reported years of SMI (missing in 314 individuals [22.1%]) and current employment status (279 [19.6%]). In Pakistan, data were complete for 166 individuals (18.0%) and missing data were reported

for years of education (722 [78.3%]), income (86 [9.3%]), years of SMI (262 [28.4%]), marital status (1 [0.11%]), employment status (33 [3.58%]) and student status (12 [1.30%]).

Bivariate results

Results from bivariate analyses can be seen in Table S1 and S2. In Bangladesh (Table S1), significant bivariate relationships with response included years of education (OR 1.03, >1.00 to 1.05), being a student (OR 2.08, 1.33 to 3.24), having a mobile phone (OR 2.31, 1.38 to 3.88) and total PHQ-9 score (OR 0.98, 0.95 to <1.00). Results from the imputed data for the two variables for which missing data were present (current employment status and years of SMI diagnosis) varied negligibly from those from the observed data.

In Pakistan (Table S2), significant bivariate relationships with response include being male (OR 1.52, 1.16 to 1.97), being formally educated (OR 1.57, 1.14 to 2.19), being employed (OR 1.47, 1.12 to 1.92) and having a mobile phone (OR 1.48, >1.00 to 2.18). Again, results on the imputed data for the six variables for which missing data were present (years of education monthly income, years of SMI, marital status, employment status and student status) varied negligibly from those on the observed data.

Table S1. Results from bivariate logistic regression analyses predicting response to Wave 1 of the COVID-19 survey in Bangladesh.

**Bangladesh**	**Odds ratio (95% CI)**	**Odds ratio (95% CI)**
	**Observed data**	**Imputed data**
**Male**	1.14 (0.92 to 1.41)	N/A
	p=0.24	
**Age**	0.99 (0.98 to >1.00)	N/A
	p=0.11	
**Years of education**	1.03 (>1.00 to 1.05)	N/A
	p=0.03	
**Formal education received**	1.33 (0.95 to 1.88)	N/A
	p=0.10	
**University educated**	0.96 (0.64 to 1.45)	N/A
	p=0.87	
**Currently employed**	1.13 (0.88 to 1.45)	1.05 (0.83 to 1.33)
	p=0.35	p=0.66
**Current student**	2.08 (1.33 to 3.24)	N/A
	p=0.001	
**Number of adult housemates**	0.97 (0.91 to 1.04)	N/A
	p=0.44	
**Currently married**	1.02 (0.82 to 1.26)	N/A
	p=0.89	
**Income per month**	1.00 (<1.00 to >1.00)	N/A
	p=0.24	
**Landline phone**	1.02 (0.36 to 2.89)	N/A
	p=0.96	
**Mobile phone**	2.31 (1.38 to 3.88)	N/A
	p=0.001	
**Years SMI**	0.99 (0.97 to 1.01)	0.99 (0.97 to 1.01)
	p=0.34	p=0.38

**PHQ-9 score**	0.98 (0.95 to <1.00)<br>p=0.05	N/A
**GAD-7 score**	0.99 (0.96 to 1.01)<br>p=0.35	N/A
**EQ5D-VAS**	1.00 (0.99 to 1.01)<br>p=0.56	N/A
**Inpatient at time of IMPACT survey**	0.89 (0.69 to 1.15)<br>p=0.36	N/A
**MINI diagnosis: major depressive v psychotic**	1.08 (0.66 to 1.77)<br>p=0.75	N/A
**MINI diagnosis: bipolar v psychotic**	1.02 (0.81 to 1.28)<br>p=0.88	N/A
**MINI diagnosis: major depressive v bipolar**	1.06 (0.64 to 1.77)<br>p=0.81	N/A

Note. The *N* in the presented bivariate analyses is 1422, except for years of SMI (n=1108) and current employment status (n=1143). Odds ratios on imputed data are presented only when that variable had missing data and thus was imputed. *CI* Confidence interval.

Table S2. Results from bivariate logistic regression analyses predicting response to Wave 1 of the COVID-19 survey in Pakistan.

Pakistan	Odds ratio (95% CI)<br>Observed data	Odds ratio (95% CI)<br>Imputed data
**Male**	1.52 (1.16 to 1.97)<br>p=0.002	N/A
**Age**	1.00 (0.99 to 1.01)<br>p=0.50	N/A
**Years of education**	0.99 (0.94 to 1.05)<br>p=0.82	1.01 (0.97 to 1.04)<br>p=0.61
**Formal education received**	1.57 (1.14 to 2.19)<br>p=0.01	N/A
**University educated**	1.33 (0.90 to 1.96)<br>p=0.15	N/A
**Currently employed**	1.47 (1.12 to 1.92)<br>p=0.01	1.43 (1.09 to 1.86)<br>p=0.01
**Current student**	1.16 (0.47 to 2.88)<br>p=0.75	1.15 (0.46 to 2.86)<br>p=0.76
**Number of adult housemates**	1.02 (0.97 to 1.08)<br>p=0.38	N/A
**Currently married**	0.95 (0.73 to 1.23)<br>p=0.71	0.95 (0.73 to 1.23)<br>p=0.69
**Income per month**	>1.00 (<1.00 to >1.00)<br>p=0.17	>1.00 (<1.00 to >1.00)<br>p=0.17
**Landline phone**	0.90 (0.60 to 1.33)<br>p=0.59	N/A
**Mobile phone**	1.48 (>1.00 to 2.18)<br>p=0.05	N/A
**Years SMI**	1.01 (0.99 to 1.02)<br>p=0.51	>1.00 (0.99 to 1.02)<br>p=0.71

**PHQ-9 score**	1.00 (0.98 to 1.01)<br>p=0.61	N/A
**GAD-7 score**	1.01 (0.98 to 1.04)<br>p=0.48	N/A
**EQ5D-VAS**	1.00 (<1.00 to >1.00)<br>p=0.99	N/A
**Inpatient at time of IMPACT survey**	1.55 (0.93 to 2.60)<br>p=0.10	N/A
**MINI diagnosis: major depressive v psychotic**	1.04 (0.68 to 1.58)<br>p=0.87	N/A
**MINI diagnosis: bipolar v psychotic**	1.16 (0.76 to 1.78)<br>p=0.49	N/A
**MINI diagnosis: major depressive v bipolar**	0.89 (0.56 to 1.32)<br>p=0.41	N/A

Note. The *N* in the presented bivariate analyses is 922, except for years of education (n=200), years of SMI (n=660), monthly income (n=836), employment status (n=889), student status (n=910) and marital status (n=921). Odds ratios on imputed data are presented only when that variable had missing data and thus was imputed. *CI* Confidence interval.

Multivariable models

Results from the final, multivariable model predicting response in Wave 1 in Bangladesh can be seen in Table S3. Only students (OR 1.93, 1.21 to 3.04) and owning a mobile phone (OR 2.21, 1.31 to 3.71) were independent predictors of response to Wave 1 of the COVID-19 survey. The AUC for this model is 0.57 (0.54 to 0.60). As no missing data was present in those variables included in the multivariable model, no sensitivity analysis on the imputed data was performed.

Table S3. Results from a multivariable logistic regression model predicting response to Wave 1 of the COVID-19 survey in Bangladesh (N= 1422).

**Bangladesh**	**Odds ratio (95% CI)**
**Years of education**	1.01 (0.99 to 1.04)
	p=0.30
**Current student**	1.93 (1.21 to 3.04)
	p=0.01
**Mobile phone**	2.21 (1.31 to 3.71)
	p=0.003
**PHQ-9 score**	0.98 (0.96 to 1.00)
	p=0.10

Note. CI Confidence interval.

In Pakistan (Table S4), owning a mobile phone (OR 1.52, 1.01 to 2.28) is the only independent predictor of response to Wave 1 of the COVID-19 survey in Pakistan. The AUC for this model is 0.58 (0.54 to 0.62). When fitting this model to imputed data (Table S4), no predictor retains statistical significance at the p=0.05 level.

Table S4. Results from a multivariable logistic regression model predicting response to Wave 1 of the COVID-19 survey in Pakistan. Coefficients are displayed on both non-imputed data (N=889) and imputed data (N=922).

**Pakistan**	**Odds ratio (95% CI)**<br>**Observed data**	**Odds ratio (95% CI)**<br>**Imputed data**
Male	1.26 (0.90 to 1.78)	1.29 (0.92 to 1.80)
	p = 0.18	p = 0.14
Educated ever	1.41 (<1.00 to 2.00)	1.36 (0.97 to 1.92)
	p = 0.05	p = 0.08
Currently employed	1.26 (0.91 to 1.76)	1.21 (0.87 to 1.69)
	p = 0.17	p = 0.26
Mobile phone	1.52 (1.01 to 2.28)	1.47 (0.99 to 2.19)
	p=0.04	p=0.06

Note. CI Confidence interval.

Discussion

This work examined the relationship between a variety of socio-demographic and health variables collected at the baseline IMPACT survey and response to Wave 1 of the COVID-19 survey. The response rates to this wave of this survey were 59.4% in Bangladesh and 49.2% in Pakistan compared to the pre-pandemic IMPACT survey. However, we observed few significant associations between the predictor variables and response. In both countries, those who owned a mobile phone were independently more likely to respond to the COVID-19 survey. Additionally, in Bangladesh, students were more likely to respond to the survey. No other variables achieved statistical significance at the p=0.05 level on multivariable analysis.

As the COVID-19 survey was carried out over the telephone, it is perhaps not a surprise that those who owned a mobile phone were more likely to respond to this survey. It can also be hypothesised that students may have more time available to contribute to research than those not a student, many of whom would be working. We observed few relationships between either health or economic factors and response. Those with a higher PHQ-9 score were less likely to respond to this survey in Bangladesh on bivariate analysis,

but this relationship vanished after adjustment. This lack of relationship between mental health and response could be because all participants in the IMPACT survey were diagnosed with SMI.

The AUCs of both models were low – 0.57 and 0.58 respectively. As a model with an AUC of 0.5 does not differentiate between responders and non-responders, we can conclude that the models built here are poor at predicting response. Our findings therefore suggest minimal differences in response propensity in this wave of the COVID-19 survey given available information at baseline. Of course, we cannot rule out that the response propensity differed due to data not available or considered in this modelling process. We included only those variables (k=16) for which we could derive a plausible hypothetical relationship with response. This inability to develop response propensity models which are capable of discriminating between responders and non-responders suggests that survey weights should not be utilised in this study or future studies utilising data from Wave 1 of the COVID-19 survey. However, the COVID-19 survey is a multi-wave survey, all drawing respondents from the cohort of individuals in the IMPACT survey. As such it will be important to repeat these analyses for subsequent waves of the COVID-19 survey.

References

1. Zavala GA, Prasad-Muliyala K, Aslam F, Barua D, Haidar A, Hewitt C, et al. Prevalence of physical health conditions and health risk behaviours in people with severe mental illness in South Asia: protocol for a cross-sectional study (IMPACT SMI survey). BMJ open. 2020;10(10):e037869.
2. Kroenke K, Spitzer RL, Williams JB. The PHQ-9: validity of a brief depression severity measure. Journal of general internal medicine. 2001;16(9):606-13.
3. Löwe B, Decker O, Müller S, Brähler E, Schellberg D, Herzog W, et al. Validation and standardization of the Generalized Anxiety Disorder Screener (GAD-7) in the general population. Medical care. 2008:266-74.
4. Janssen M, Pickard AS, Golicki D, Gudex C, Niewada M, Scalone L, et al. Measurement properties of the EQ-5D-5L compared to the EQ-5D-3L across eight patient groups: a multi-country study. Quality of Life Research. 2013;22(7):1717-27.
5. StataCorp L. Stata statistical software. College Station TX. 2009.
